# An SPRI beads-based DNA purification strategy for flexibility and cost-effectiveness

**DOI:** 10.1186/s12864-023-09211-w

**Published:** 2023-03-16

**Authors:** Danli Liu, Qiujia Li, Jing Luo, Qitong Huang, Yubo Zhang

**Affiliations:** 1 Shenzhen Branch, Guangdong Laboratory of Lingnan Modern Agriculture, Key Laboratory of Livestock and Poultry Multi-omics of MARA, Agricultural Genomics Institute at Shenzhen, Chinese Academy of Agricultural Sciences, 7 Pengfei Road, Dapeng, Shenzhen, 518120 China; 2 Shenzhen Branch, Guangdong Laboratory for Lingnan Modern Agriculture, Genome Analysis Laboratory of the Ministry of Agriculture, Agricultural Genomics Institute at Shenzhen, Chinese Academy of Agricultural Sciences, Shenzhen 518120, China; 3 Animal Breeding and Genomics, Wageningen University & Research, Wageningen, 6708PB, Netherlands; 4 College of Life Science and Engineering, Foshan University, Foshan, China

**Keywords:** PEG 8000, SPRI beads, DNA purification method, DNA fragments selective

## Abstract

**Background:**

Current solid-phase reversible immobilization (SPRI) beads technology is widely used in molecular biology due to its convenience for DNA manipulation. However, the high performance commercial SPRI beads have no price advantage over our method. Furthermore, the use of commercially available SPRI beads standards does not provide the flexibility required for a number of specific nucleic acid handling scenarios.

**Results:**

We report an efficient DNA purification strategy by combining home-made beads-suspension buffer with SPRI beads. The method tests the critical concentrations of polyethylene glycol (PEG) 8000 and beads to maximise recovery. And the composition of the SPRI beads DNA purification system (SDPS) was determined at 20% PEG 8000, 2 M NaCl and 16.3 mM MgCl_2_, and 1.25 mg/ml beads (1/8th original concentration). Then, we tested the DNA recovery of the SDPS, and the result showed that it was comparable to the control (AMPure XP beads). In the study, we have also developed an adjustment SPRI beads DNA purification system (ASDPS), the volume of ASDPS per reaction is 0.6× reaction volume (beads/samples). The performance of ASDPS is similar to SDPS and the control. But the cost of our methods is only about 1/24th of the control. To further assess its performance, we prepare the DNA-seq libraries to evaluate the yield, library quality, capture efficiency and consistency. We have compared all these results with the performance of the control and confirmed its efficiency.

**Conclusion:**

We have proposed an alternative DNA purification approach with great flexibility, allowing researchers to manipulate DNA in different conditions. And ultimately, its application will benefit molecular biology research in the future.

**Supplementary Information:**

The online version contains supplementary material available at 10.1186/s12864-023-09211-w.

## Background

Currently, the use of solid-phase reversible immobilization (SPRI) paramagnetic beads is fundamental to the extraction and manipulation of nucleic acids (DNA & RNA) [1–4]. For example, SPRI beads have been used in the clean-up and size-selection (150-800 bp) of next-generation sequencing (NGS) libraries [5]; Optimised SPRI beads-based purification system could be used as an alternative method to recover the longer DNA fragments (>6kbp) in the third generation sequencing methods [6, 7]; SPRI beads are also used to manipulate the target DNA fragments in the three-dimensional genomics technologies such as high-through chromosome conformation capture (Hi-C) (reverse crosslinking and DNA purification) [8], in situ Hi-C (DNA shearing and size selection) [9], and Exo-Hi-C (marking of DNA ends and blunt-end ligation and DNA purification) [10]. Compared to traditional DNA extraction methods that rely on silica columns or organic solvents, SPRI beads technology could avoid many tedious steps and toxic chemicals [11–13]. In addition, SPRI beads are also simple, efficient, and high-throughput and more suitable for automated platforms [14]. However, the typical application of commercial beads has been limited to the ‘standard’ reaction volume of target DNA fragments. In addition, there is a need for the flexibility in some specific DNA manipulation scenarios, i.e., the DNA content is fixed but the sample volume is large.

The SPRI bead DNA purification method can be optimized by adjusting the concentration of polyethylene glycol (PEG) and NaCl/MgCl_2_ [5, 6, 15, 16]. The principle of this technique is that PEG could induce the coil-to-globule conformational transition of DNA, exposing a large number of negatively charged phosphate groups on the phosphate skeleton and binding to negatively charged functional group-modified polymer paramagnetic particles (i.e. carboxyl) on the surface [15]. And PEG could increase the solution’s viscosity, keeping the beads suspended rather than settling [17]. Meanwhile, NaCl/MgCl_2_ provides ‘salty ion bridging’ of negatively charged molecules, allowing the exposed phosphate group of the DNA to bind to the carboxyl group of the beads [18]. DNA recovery is also affected by the performance of SPRI beads (surface chemistry, diameter, shape, and magnetic properties) [19–22]. In addition, the recovery of SPRI beads-based methods is related to the binding threshold of and the DNA beads (DNA size > 150 bp) [23, 24]. These findings help to adjust the SPRI bead-based DNA purification approach to meet different experimental needs. Based on this, we are trying to develop an effective strategy for DNA manipulation. It can be used effectively in any DNA manipulation scenario and the reaction volume can be adjusted to accommodate high working ratios or the sample of the fixed DNA content (relatively low) with large-volume.

In this study, we present a strategy for DNA extraction and manipulation of the SPRI beads DNA purification method, an alternative method that combines SPRI beads and homemade beads-suspension buffer. Firstly, the factors determined for the SPRI beads DNA purification system (SDPS) are described in detail. The optimal concentrations of PEG 8000 and the beads were determined in the present condition of NaCl/MgCl_2_. We have also developed an adjustment reaction volume ratio SPRI beads DNA purification system (ASDPS). The ASDPS works as 0.6× (beads/sample), the novelty being that the 0.6× is equivalent to the conventional 1.2×, which is suitable for large volume samples and can be flexible to manipulate DNA in different conditions. Next, we investigate the recovery of DNA fragments of the SDPS methods compared to the control (AMPure XP beads), and the fragment selection of different reaction volumes of the SDPS. Finally, we evaluate the empirical performance of SDPS in the purification/clean-up steps of the DNA library preparation workflow. Our results showed that the SDPS methods were adjustment, high-performance, and cost-effictive for DNA extraction and manipulation and were generally applicable for genomic applications. The significance is that it provides users with an option to economical and flexible way to use SPRI beads for DNA purification.

## Results

### Determining of PEG and beads concentrations for SDPS

**Fig. 1:**
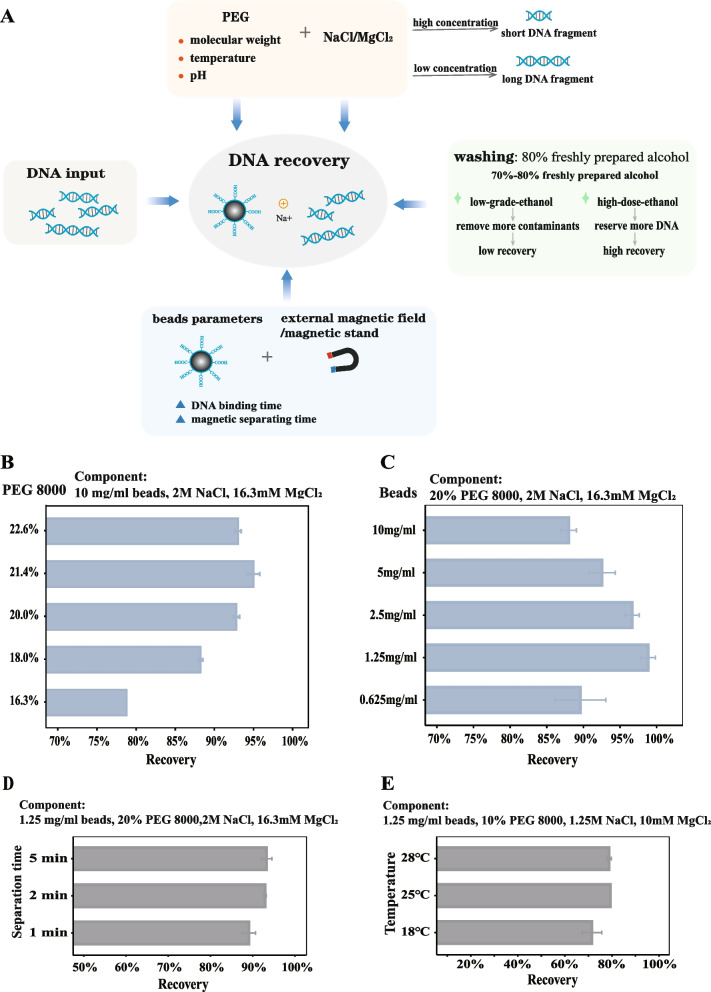
The determining factors of the SPRI beads-based DNA recovery. **A** The factors affecting the DNA recovery in bead-based DNA purification method. **B** The DNA recovery with different PEG 8000 concentrations. **C** The DNA recovery with different particle concentrations. **D** The DNA recovery with different separation time on magnetic rack. **E** The DNA recovery with different temperature on DNA-particle binding

We first analysed the factors that make the SPRI beads-based purification system work, including beads parameters, the PEG/NaCl/MgCl_2_, etc. (Fig. 1A). Based on previous studies that high-molecular-weight PEG, such as PEG 8000, is more likely to induce DNA collapse than low-molecular-weight PEG [25], we chose to use PEG 8000 in the beads-suspension buffer. we then investigate these two key parameters, the concentration of PEG 8000 and SPRI beads, which together determine the efficiency of DNA recovery. We tested the DNA recovery of different fractions of the beads-suspension buffer to determine the optimal purification system. The results indicate that the recovery rate of buffer 4, which contains 16.3% PEG 8000, 2 M NaCl, and 16.3 mM MgCl_2_, was significantly higher compared to that of buffer1, buffer2, and buffer3 (*p* < 0.05) (Fig. S1). Based on this, we determined the salt concentration (2 M NaCl and 16.3 mM MgCl_2_) of the SDPS. We then tested the concentration of PEG 8000 from 16.3 to 22.6%, while keeping the other basic components remained in the same condition (2 M NaCl, 16.3 mM MgCl_2_ and 10 mg/mL beads). When the concentration of PEG 8000 was increased from 16.3 to 20%, the recovery increased from 78.65 to 92.42% ± 0.75% (*p* < 0.05) (Fig. 1B, Table 1). Our choice of 20% PEG was consistent with the results of previous studies [3]. Next, the relation of the critical of beads concentration to DNA recovery and beads-suspension buffer was studied. We tested the different concentrations of beads in the beads-suspension buffer (20% PEG, 2 M NaCl, and 16.3 mM MgCl_2_, pH = 5.1). We found that as the beads concentration decreased from 10 mg/ml to 1.25 mg/ml, the recovery increased inversely. The recovery was highest at 1.25 mg/ml (*p* > 0.05) (Fig. 1C, Table 1). We assumed that PEG 8000 would increase the viscosity of the buffer, slow down the precipitation of the beads and increase the binding of the DNA-beads within a fixed 10 minutes [17]. In addition, the magnetic separation time had little effect on recovery efficiency when longer than 1 minute (*p* > 0.05) (Fig. 1D, Table 1). The workflow of this protocol was advised to operate at room temperature (25 °C) (Fig. 1E, Table 1). Therefore, we proposed an efficient SDPS (1.25 mg/ml beads and beads-suspension buffer: 20% PEG, 2 M NaCl, and 16.3 mM MgCl_2_) for DNA extraction at 1.2× reaction volume, and the magnetic separation time was reduced to 2 minutes.

### Assessing the performance of SDPS

**Fig. 2:**
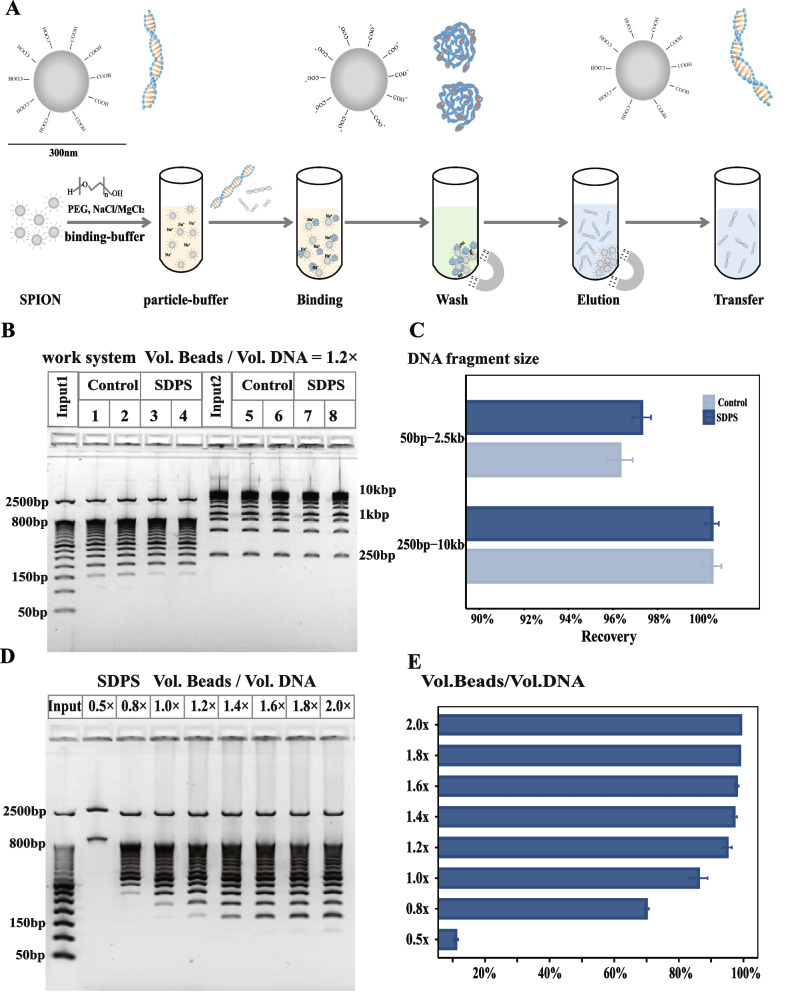
Establishment and assessment of the SDPS method. **A** Workflow illustration of SPRI beads-based DNA extraction. **B** and **C** E-Gel visualization and recovery of DNA extracted using SDPS at different DNA size range markers. Control: AMPure XP beads, Input 1: 50 bp DNA marker (50 bp–800 bp, 2500 bp); Input 2: 1kbp DNA marker (250 bp-10 kb)；each group had four replicates, Mean ± SD. **D** and **E** Absorbing range and recovery of SDPS with different working ratio. Input: 50 bp DNA marker (50 bp–800 bp, 2500 bp); each group had two replicates, Mean ± SD. Figure 2B and D were cropped and the original gels are presented in Supplementary file 2 Fig. S2 and Fig. S3

To test whether the performance of the SDPS could be an achievable standard, we prepared the AMPure XP bead as a control standard. The SDPS method workflow is described in Fig. 2A. For input1 (50 bp–2500 bp), it is apparent that recovery of DNA fragments larger than 150 bp (Fig. 2B), the control and SDPS recovered 95.80 ± 1.41% and 96.80 ± 1.00% DNA, respectively (*p* > 0.05) (Fig. 2C). However, approximately 5% of DNA fragments were lost due to DNA fragments smaller than 150 bp. In comparison, the control and SDPS recovered almost 100% DNA in the range of 250 bp to 10kbp (*p* > 0.05) (Fig. 2C). There was no significant difference between the control group and SDPS (*p* > 0.05) (Fig. 2B, C). Therefore, these results indicate that SDPS can efficiently extract DNA in a wide range from 150 bp to 10kbp, and the recovery was not significantly different from the control.

We have also tested the selective precipitation of DNA fragments from the SDPS method using different reaction volume ratios (Fig. 2D, E). 0.5× (Vol. Sample: Vol. Bead = 1: 0.5, the same as below) to eliminate fragments < 800 bp, 0.8×, 1× and 1.2× to recover DNA fragments of 250 bp, 200 bp and 150 bp, respectively. 1.6 × − 2.0× to recover DNA fragments > 100 bp (*p* > 0.05) (Fig. 2D). The results showed that SDPS allows for efficient size selection purification depending on the sample/bead volume ratio used.

### ASDPS is an adjustable reaction volume ratio method for DNA purification

Currently, the target DNA (> 150 bp) is required to be at least 1.2 times or even 2 times the beads/sample ratio. To address the limitations of the standard reaction volume of SPRI beads technology. We turned to the strategy of reducing the buffer volume by half, but using the same composition of PEG 8000, beads, NaCl and MgCl_2_ in the DNA-beads mixture as in SDPS. We have therefore developed an alternative ASDPS method using 1.818 mg/ml SPRI beads and beads-suspension buffer: 29.09% PEG, 2.909 M NaCl and 23.709 mM MgCl_2_ (pH = 3.8). The volume ratio of beads to DNA samples of ASDPS is 0.6 times, i.e. the 0.6× reaction volume is equivalent to the conventional 1.2× reaction volume.

**Fig. 3:**
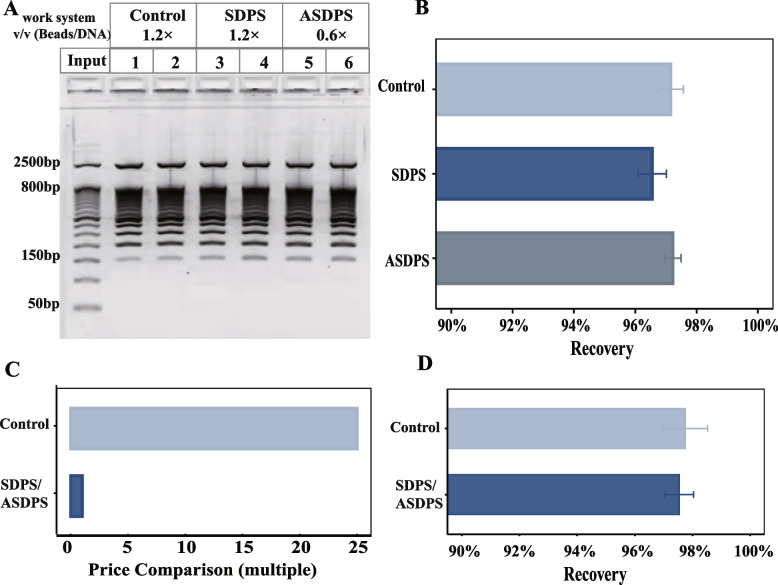
Performance evaluation of the SDPS methods. **A** E-Gel visualization of DNA extracted using the Control, SDPS and ASDPS. **B** The DNA recovery of the control, SDPS and ASDPS. Input: 50 bp DNA marker (50 bp–800 bp, 2500 bp), each group had four replicates, Mean ± SD. **C** The price comparison between control and SDPS/ASDPS. **D** Comparison of recovery between the control and SDPS/ASDPS, each group had at least eight replicates, Mean ± SD. Figure 3**A** was cropped and the original gels is presented in Supplementary file 2 Fig. S4

Next, we evaluated the empirical performance of ASDPS compared to SDPS and control. DNA fragment size recovery was highly consistent between control, SDPS and ASDPS (Fig. 3A). The DNA recovery of the control, SDPS and ASDPS was 97.15 ± 0.72%, 96.55 ± 0.93% and 97.23 ± 0.54%, respectively (*p* > 0.05) (Fig. 3B). Therefore, there is no significant difference in the DNA recovery between the control and SDPS methods (*p* > 0.05) (Fig. 3D, Table 2). Moreover, the SDPS methods are extremely cost-effective, costing only about 1/24 of the control (Fig. 3C, Table 2). In summary, our SDPS methods are efficient, economical, and adjustable methods for DNA purification.

**Table 1:** Compare the performance and cost of the Control, SPDS, and ASPDS

	Recovery(%) Mean ± SD	Range	Working system	Cost	Price advantage (XP/SDPS)
Control	97.75 ± 2.06%	150 bp-10kbp	1.2×	Fr. 80.15–91.6/ml	about 24 times
SDPS	97.70 ± 1.97%	150 bp-10kbp	1.2×	￥109.2–212/ml	
ASDPS	97.23 ± 0.05%	150 bp-10kbp	0.6×		

Control is commercial AMPure XP beads. Prices are from the official website:

https://www.beckmancoulter.com/en/products

https://www.beckman.com/reagents/genomic/cleanup-and-size-selection/pcr#

http://www.beaverbio.com/products/view/1287.html

**Table 2:** Experiment tested

Test component	Buffer composition	Work condition			
		Vol.beads/Vol.DNA	Separating time (min)	Incubated temperature	Incubated time (min)
PEG 8000	16.3% PEG 8000, 10 mg/ml beads, 2 M NaCl, 16.3 mM MgCl_2_	1.2×	5	RT	10
	18% PEG 8000, 10 mg/ml beads, 2 M NaCl, 16.3 mM MgCl_2_	1.2×	5	RT	10
	20% PEG 8000, 10 mg/ml beads, 2 M NaCl, 16.3 mM MgCl_2_	1.2×	5	RT	10
	21.4% PEG 8000, 10 mg/ml beads, 2 M NaCl, 16.3 mM MgCl_2_	1.2×	5	RT	10
	22.6% PEG 8000, 10 mg/ml beads, 2 M NaCl, 16.3 mM MgCl_2_	1.2×	5	RT	10
Beads (SPION particles)	10 mg/ml beads, 20% PEG 8000, 2 M NaCl, 16.3 mM MgCl_2_	1.2×	5	RT	10
	5 mg/ml beads, 20% PEG 8000, 2 M NaCl, 16.3 mM MgCl_2_	1.2×	5	RT	10
	2.5 mg/ml beads, 20% PEG 8000, 2 M NaCl, 16.3 mM MgCl_2_	1.2×	5	RT	10
	1.25 mg/ml beads, 20% PEG 8000, 2 M NaCl, 16.3 mM MgCl_2_	1.2×	5	RT	10
	0.675 mg/ml beads, 20% PEG 8000, 2 M NaCl, 16.3 mM MgCl_2_	1.2×	5	RT	10
Separating time	1.25 mg/ml beads, 20% PEG 8000, 2 M NaCl, 16.3 mM MgCl_2_	1.2×	5	RT	10
	1.25 mg/ml beads, 20% PEG 8000, 2 M NaCl, 16.3 mM MgCl_2_	1.2×	2	RT	10
	1.25 mg/ml beads, 20% PEG 8000, 2 M NaCl, 16.3 mM MgCl_2_	1.2×	1	RT	10
Incubated temperature	1.25 mg/ml beads, 10% PEG 8000, 1.25 M NaCl, 10 mM MgCl_2_	1.2×	5	28 °C	10
	1.25 mg/ml beads, 10% PEG 8000, 1.25 M NaCl, 10 mM MgCl_2_	1.2×	5	25 °C	10
	1.25 mg/ml beads, 10% PEG 8000, 1.25 M NaCl, 10 mM MgCl_2_	1.2×	5	18 °C	10

### The SDPS methods worked well with sequencing libraries

**Fig. 4:**
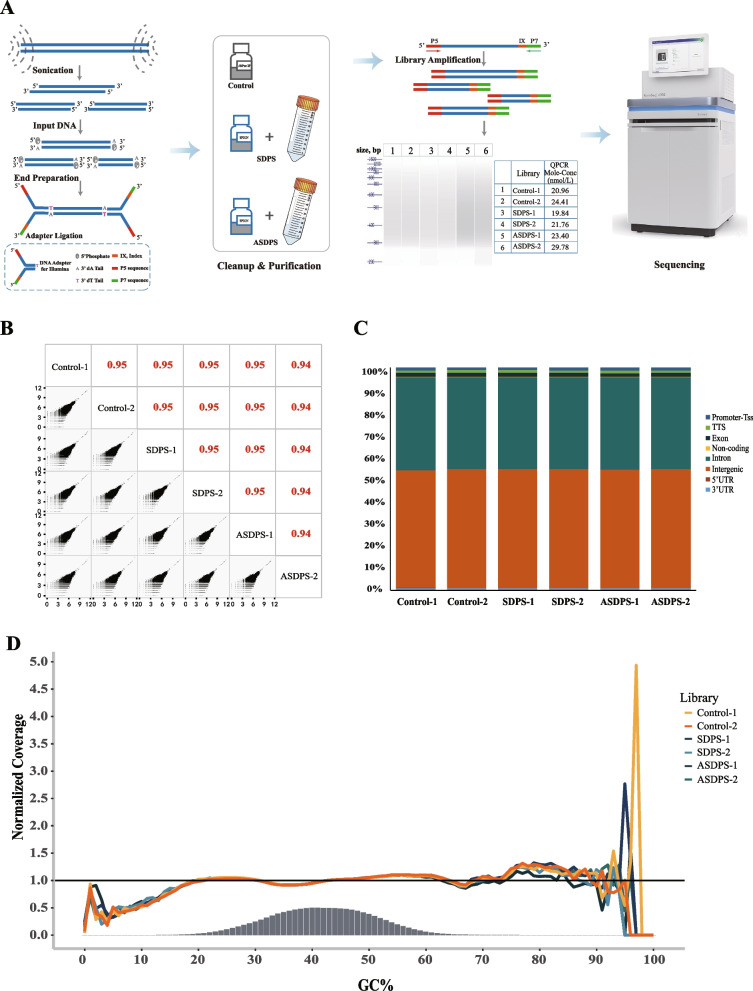
Schematic overview of the library preparation and data analysis. **A** Experimental workflow of the library preparation procedure using the Control, SDPS and ASDPS. **B** Read depth correlation shows consistently high coverage among these libraries of the Control, SDPS and ASDPS. Coverage of each 10 kb region of mm9 (as determined by bedtools coverage) was compared among Control, SDPS and ASDPS. Most regions are covered by ~ 1000 reads, as expected. Low and high coverage regions are well correlated. Pearson correlation values among each pair of comparisons, with the size of the text proportional to the magnitude of the correlation coefficient (upper diagonal elements). **C** Histogram of Reads distribution in different regions of the genome. Each square bar in the figure represents a library, the height of each region represents the percentage of Mapped Reads in all Mapped Reads Mapped to this region. **D** GC coverage of the six DNA libraries. Expected normalized coverage of 1.0 is indicated by the horizontal grey line, the number of 100 bp regions at each GC% is indicated by the vertical grey bars, and the colored lines represent the normalized coverage for each library

An essential step in NGS library preparation is the use of SPRI beads for clean-up and size selection. To test whether SDPS methods can meet the stringent needs of today’s genomic applications and don’t lose critical data for NGS sequence libraries when sequenced on the Illumina® platform from Novogene Corporation. We prepared the DNA libraries using the 3 cleanup steps (control, SDPS, and ASDPS) to remove contaminants, such as: adapter, enzyme, PCR primer, dNTPs or adapter dimers. These libraries passed the company’s quality control and the fragment sizes of the library were almost consistent (Fig. 4A). Then, the unique mapped paired-end reads from the clean reads were mapped to the mouse genome. The mapped ratio of these libraries ranges from 97.01 to 97.43%, obtained with a Phred quality value > 30 occupying 88.58–92.59%, the range of GC content is about 41.90%. After the final removal of reads with duplicate sequences due to PCR amplification artifacts, the unique mapped ratio of these libraries ranges from 78.22 to 78.63% (Table 3). The yield of these libraries differed, but the data obtained still met the requirements for informative analysis and ensured the reliability of downstream data analysis. Furthermore, we evaluated the GC content and correlation coefficient of the six DNA libraries. The GC coverage and composition were compared with the SDPS groups and the control, and the results show that the SDPS groups are the most similar to the libraries of the control group libraries and also cover the range of GC content (Fig. 4D). The number of Mapped Reads in different regions of the specified reference genome (5’UTR, promoter-TSS, TTS, exon, non-coding, intron, intergenic, 3’UTR) is shown in Fig. 4C. Comparison of the level of sequence coverage, at 10 kbp intervals, these libraries showed good correlation (0.94–0.95) (Fig. 4B).

**Table 3:** The sequencing data statistics of SDPS, ASDPS, and Control

Library	Total reads	Mapped reads	Mapped ratio (%)	Unique mapped reads	Unique mapped ratio(%)	Peak size(bp)	Q30(%)	GC Content(%)
Control1	18,202,392	17,730,474	97.41	14,296,198	78.54	478	89.36	41.86
Control2	20,230,356	19,693,251	97.35	15,887,539	78.53	477	88.88	41.94
SDPS1	15,500,782	15,102,483	97.43	12,187,960	78.63	465	92.95	41.91
SDPS2	23,057,956	22,412,946	97.20	18,071,427	78.37	474	89.03	41.88
ASDPS1	18,090,910	17,549,250	97.01	14,150,876	78.22	479	88.58	41.85
ASDPS2	10,793,584	10,491,316	97.20	8,464,116	78.42	472	93.44	42.00

Total reads: number of clean reads, by single end; mapped reads: the number of reads compared to the reference genome; mapped ratio: the percentage of clean reads that are mapped into the reference genome; unique mapped reads: the number of reads mapped to a unique location in the reference genome; unique mapped ratio: the percentage of clean reads that are mapped to a unique location in the reference genome

We empirically show that library cleanup using our methods is a feasibility choice for analyses requiring very high analytical stringency. SDPS methods are at least as efficient as widely used commercial alternatives while maintaining the cost advantage and quality of purity DNA. As a result, our methods provide high-performance isolation, purification and clean-up protocols that support applications such as qPCR, ddPCR, Sanger sequencing, NGS and microarrays.

## Discussion

In this study, we developed effective and economical methods for the extraction and purification of DAN: SDPS and ASDPS. We have determined that the optimal operating system for SDPS is 20% PEG8000, 2 M NaCl, 16.3 mm MgCl_2_, and 1.25 mg/ml beads by testing critical PEG8000 and beads concentrations. ASDPS was then developed by halving the volume of the beads-suspension buffer but maintaining the same final concentration as the components of SDPS. We further evaluated SDPS and ASDPS for recovery efficiency, recovered DNA fragment size, and fragment size selection for different reaction volumes of SDPS. The SDPS and ASDPS show similar experimental performance compared to the control (AMPure XP beads). The ASDPS requires only 0.6× reaction volume for DNA manipulation, which is excellent convenience for experimental operators. In addition, we evaluated the performance of the SDPS methods in terms of recovery, DNA fragment size selection, and library construction and clean-up compared to the gold standard AMPure XP beads, demonstrating its applicability and efficiency in the purification and manipulation of DNA.

The economic benefits of using SDPS methods are not only relevant to DNA manipulation such as extraction, size-selection, and library clean-up, but will also save on beads resources - only 1/8 of the raw material (SPRI beads). At approximately 24 times less expensive than AMPure XP beads, SDPS methods are suitable for large-scale DNA extraction experiments or high-throughput DNA manipulation on a budget [5, 26]. In addition, our methods provide an alternative to replace ethanol precipitation for recovering DNA samples (> 150 bp) with fixed DNA content but large-volume.

A striking feature of the ASDPS is its adaptability and universality. The ASDPS can replace the standard 1.2× reaction volume, which is more suitable for DNA manipulation with high reaction volume ratio or large-volume samples. For example, in our laboratory, in our Hi-C process, after the step of marking DNA ends and blunt-end ligation, the sample volume is about 800ul, in the following DNA purification process, our methods can avoid the lack of matching magnetic frame or complicated handling operation due to the large total volume, thus reducing unnecessary sample loss and increasing recovery efficiency [10]. In addition, the feature of reducing the total volume of samples may help to avoid tedious handling operations, or mismatched magnetic frames, and minimize cross-contamination between different samples using the 96-well for automated instruments [27–29].

We offer an alternative strategy for DNA purification based on SPRI beads, allowing the researcher to adjust the concentration of the SPRI beads and make the optimal bead-suspension buffer. To maximize DNA recovery, researchers only consider the mainly decisive factors: PEG 8000 and salt concentration, beads concentration and parameters. Previous studies have focused on adjusting the concentrations of PEG and NaCl for DNA size selection, but have easily overlooked the concentration of beads in the overall purification system [16, 30, 31]. A possible limitation of our methods is that the stock buffer is not universal due to the parameters of different types of magnetic beads. However, any researcher can further optimise the bead suspension buffer for efficient recovery or for different experimental purposes according to our conditions.

## Conclusion

In summary, we have developed SDPS methods that can effectively extract and manipulate DNA in any DNA manipulation scenario, from standard commercial DNA purification method to adjusted SPRI beads concentrations and optimal beads-suspension buffer, providing researchers with a great deal of flexibility and convenience to meet different DNA manipulation conditions. Moreover, this DNA purification strategy provides an extremely economical and flexible alternative to the use of SPRI beads for nucleic acid isolation and purification, and our method has great potential for further applications in the field of biology and medicine.

## Materials and methods

### Materials

SPRI beads: carboxylated super-paramagnetic iron oxide nanoparticles (SPION) (BeaverBeads™ Mag COOH-300, Beaver, cat. #70106), Polyethylene glycol (PEG) 8000, Sigma-Aldrich, cat. # 89510-250G-F), Sodium chloride (NaCl, Thermo Fisher Scientific, cat. # AM9760G), Magnesium chloride (MgCl_2_, Thermo Fisher Scientific, cat. #AM9530G), and UltraPure™ Distilled Water (Thermo Fisher Scientific, cat. #2186760). DNA samples contains two types of DNA ladders (input1: 50 bp–2500 bp; input2: 250 bp-10kp, Thermo Fisher Scientific, cat. #10416014), plasmid DNA and mouse embryonic stem cell (mESC) genomic DNA. DNA at the range of 100 ng-1μg was used in all of our test experiments. Commercial extraction kit Agencourt® AMPure® XP (Beckman Coulter, cat. # A63880) as control group.

## Methods

### Test for the critical concentrations in beads-suspension buffer

Different buffer compositions (the critical concentration of PEG 8000 and beads) and working conditions (magnetic separation times and DNA-particle binging temperature) were tested in the beads/DNA mixture (Table 1). SPRI beads (10 mg/ml) were washed twice with distilled water and diluted 8 times with beads-suspension buffer. DNA extraction and assessment were performed as follows: 1.2× particle-buffers into each equal molecular weight DNA sample tube. Beads were washed twice with 80% ethanol and eluted in 20-40ul of DNase/RNase-free water or low-ionic-strength Tris-Ethylene Diamine Tetra acetic Acid (EDTA) buffer (TE) using an external magnetic field (magnetic stand). Yield and fragment distribution of all eluted DNA samples were assessed using the Qubit® Fluorometer dsDNA HS Assay Kit (Invitrogen) and 2% agarose E-gel® 96 precast gel (Invitrogen), respectively.

### Library preparation and sequencing

To extract the murine genomic DNA, we used the Cell/Tissue DNA Isolation Mini Kit (Vazyme Cat.DC102–01). DNA-seq libraries were prepared from mESC genomic DNA fragments using the VAHTSTM Universal DNA Library Prep Kit for Illumina® V3 Kit (cat. #ND607) and VAHTSTM DNA Adapters set3-set6 for Illumina® (cat. #N805) according to the manufacturer’s instructions. We started Bioruptor Sonication with 100 ng 200-500 bp mESC gDNA fragments, and the libraries were amplified with 5 cycles of PCR. After adapter ligation and PCR amplification, we use control (1.2×), SDPS (1.2×), and ASDPS (0.6×) to remove contaminants (dNTPs, salts, primers, primer dimers). DNA libraries were sequenced on a HiSeq4000 at Novogene, generating pair end 150-bp reads.

### Statistical analysis and DNA library analysis software

Data for testing the factors (PEG8000, SPRI beads concentration, separation time, and temperature) were evaluated using one-way ANOVA (IBM SPSS Statistics 25) with at least three replicates for each group. The other data were evaluated by the Independent-Samples T Test using SPSS software (IBM SPSS Statistics 25). Mean ± Standard deviation (Mean ± SD) is represented on diagrams. If *p* > 0.05, there was no significant difference found between the groups, as determined by the results of the one-way ANOVA or the Independent-Samples T Test. The DNA library raw sequence quality was measured using FastQC (version 0.11.7). Reads were trimmed from the adapter sequence using Cutadapt (version 1.16). Reads were mapped to the mouse genome mm9, using Bowtie2 (version 2.2.9). Duplicate sequences were removed using Samtools-rmdup (version 1.7), and the reads were annotated using Homer AnnotatePeaks.pl (version 4.11). GC content and uniform coverage per 100 bp were calculated using Picard CollectGcBiasMetrics (version 2.22.3). Quantity and coverage of reads per 10kbp were calculated using Bedtools coverage (version v2.27.0). Relationship analysis of each library was performed using R (version 4.0.2).

### Supplementary Information

**Additional file 1: Fig. S1.** The assessment of homemade bead-suspension buffers.

**Additional file 2: Supplementary file 1.** Detailed use instruction for SDPS methods.

**Additional file 3: Supplementary file 2.** The original DNA Electrophoretic in the paper.

## Data Availability

The sequences data reported in this study was archived in the Sequence Read Archive (SRA) under the accession number SRP369854 (https://www.ncbi.nlm.nih.gov/sra/?term=SRP369854) and the BioProject Accession number PRJNA824260 (https://www.ncbi.nlm.nih.gov/bioproject/?term=PRJNA824260).

## Abbreviations

PEG: Polyethylene glycol
NGS: Next-generation sequencing
SPRI: Solid-phase reversible immobilization
SDPS: SPRI beads DNA purification system
ASDPS: Adjustment SPRI beads DNA purification system
TE buffer: Tris-Ethylene Diamine Tetraacetic Acid (EDTA) buffer
