# Correction: An SPRI beads-based DNA purification strategy for flexibility and cost-effectiveness

**DOI:** 10.1186/s12864-023-09551-7

**Published:** 2023-08-10

**Authors:** Danli Liu, Qiujia Li, Jing Luo, Qitong Huang, Yubo Zhang

**Affiliations:** 1 Shenzhen Branch, Guangdong Laboratory of Lingnan Modern Agriculture, Key Laboratory of Livestock and Poultry Multi-Omics of MARA, Agricultural Genomics Institute at Shenzhen, Chinese Academy of Agricultural Sciences, 7 Pengfei Road, Dapeng, 518120 Shenzhen China; 2 Shenzhen Branch, Guangdong Laboratory for Lingnan Modern Agriculture, Genome Analysis Laboratory of the Ministry of Agriculture, Agricultural Genomics Institute at Shenzhen, Chinese Academy of Agricultural Sciences, Shenzhen, 518120 China; 3 Animal Breeding and Genomics, Wageningen University & Research, 6708PB Wageningen, Netherlands; 4 College of Life Science and Engineering, Foshan University, Foshan, China

**Correction: BMC Genomics 24, 125 (2023)**

**https://doi.org/10.1186/s12864-023-09211-w**

Following publication of the original article [1] it was reported that there was an error in Fig. 1. Panels B and C were duplicates. The correct Fig. 1 is given in this Correction and the original article has been updated.

**Fig. 1:**
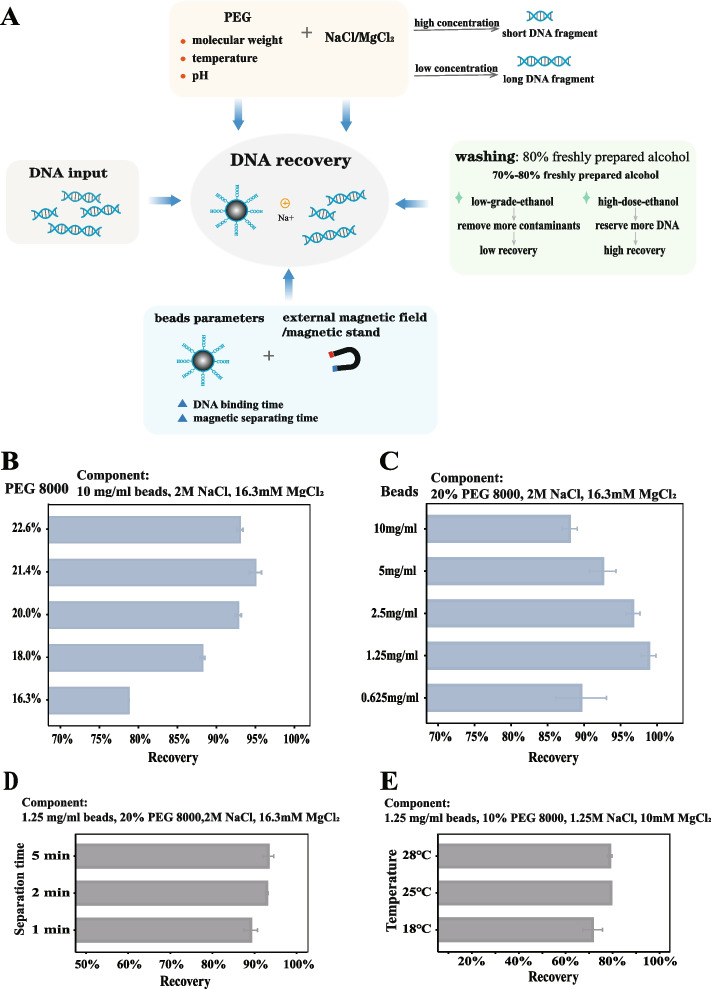
The determining factors of the SPRI beads-based DNA recovery. **A** The factors affecting the DNA recovery in bead-based DNA purification method. **B** The DNA recovery with different PEG 8000 concentrations. **C** The DNA recovery with different particle concentrations. **D** The DNA recovery with different separation time on magnetic rack. **E** The DNA recovery with different temperature on DNA-particle binding
