# Supplementary figures and images for: An SPRI beads-based DNA purification strategy for flexibility and cost-effectiveness
Source: BMC Genomics. 2023 Mar 16;24:125. doi: 10.1186/s12864-023-09211-w (PMC10022144; doi:10.1186/s12864-023-09211-w)

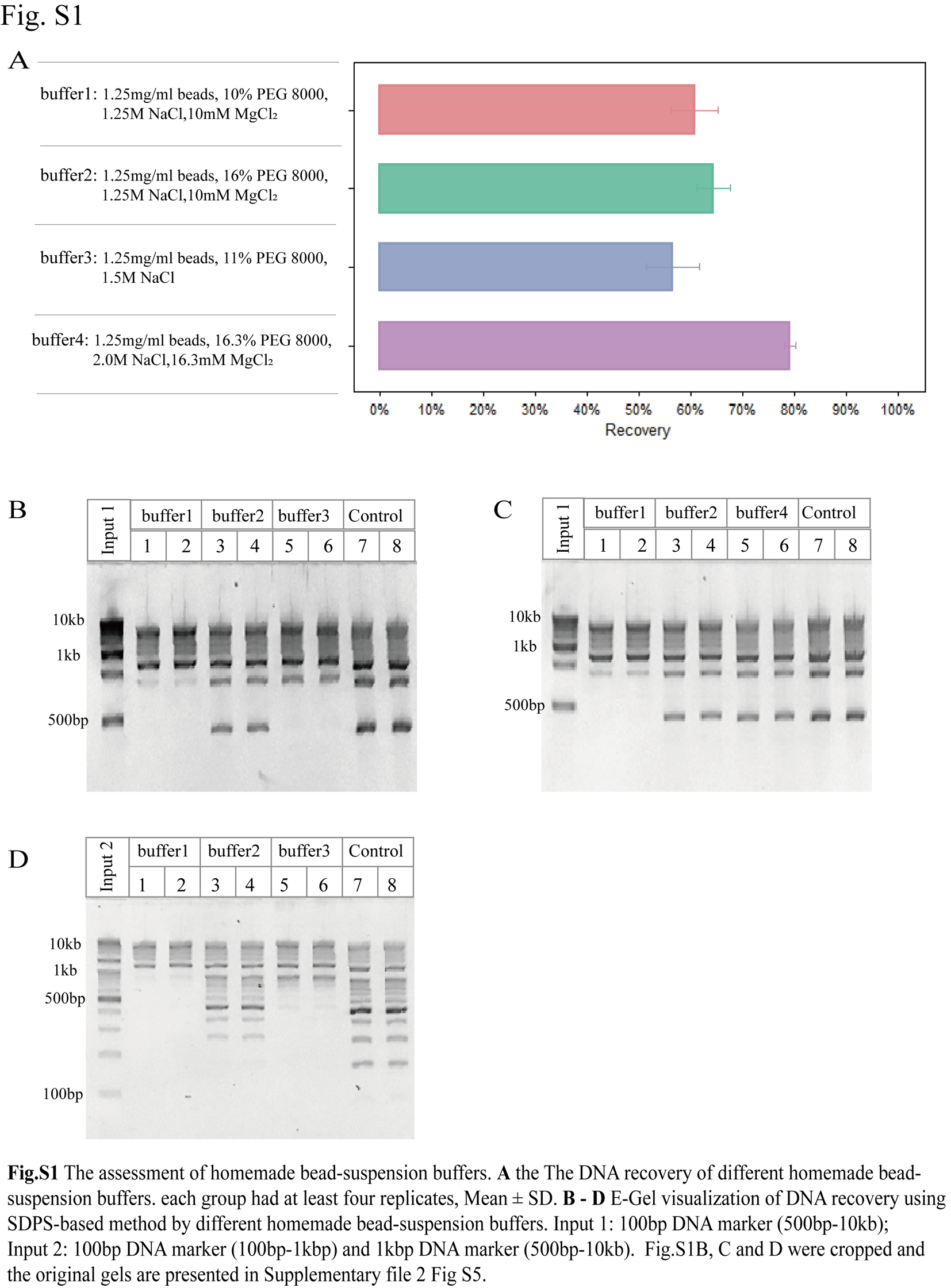

Supplement: Supplementary file 1 — Additional file 1: Fig. S1. The assessment of homemade bead-suspension buffers. [file 12864_2023_9211_MOESM1_ESM.tif]
