# Supplementary material for: An SPRI beads-based DNA purification strategy for flexibility and cost-effectiveness
Source: BMC Genomics. 2023 Mar 16;24:125. doi: 10.1186/s12864-023-09211-w (PMC10022144; doi:10.1186/s12864-023-09211-w)
Supplement: Supplementary file 2 — Additional file 2: Supplementary file 1. Detailed use instruction for SDPS methods. [file 12864_2023_9211_MOESM2_ESM.docx]

**Ready-to-use instructions**

**SDPS method**

1. Shake the BeaverBeadsTM Mag COOH-300 bottle to resuspend any magnetic particles that may have settled. Leave at room temperature for 30 min.
2. The magnetic beads were washed twice with distilled water, eg: add 100ul resuspended beads in the tube, place the tube on an appropriate magnetic stand to separate the beads from the supernatant, repeat for a total of two washes.
3. Add 800 μl buffer A (beads: buffer 10 =1:8) in the tube and mix well (800ul beads-buffer).

| Buffer A | Stock | 10mL |
| --- | --- | --- |
| PEG 8000 |  | 2g |
| NaCl | 5M | 4mL |
| MgCl2 | 2M | 163ul |
| ddH2O |  | volume to 10mL |

1. This step binds DNA fragments to the magnetic beads. Mix reagent and sample thoroughly by pipette mixing 10 times. Let the mixed samples incubate for 10 minutes at room temperature for maximum recovery. Eg: 120ul beads-buffers are added into 100ul DNA samples tubes, mix well and sit at 25 °C for 10 mins.
2. Place the tube on an appropriate magnetic stand to separate the beads from the supernatant. If necessary, quickly spin the sample to collect the liquid from the sides of the tube or plate wells before placing on the magnetic stand.
3. Add 200 μl of 80% freshly prepared ethanol to the tube while in the magnetic stand. Incubate at room temperature for 30 seconds, and then carefully remove and discard the supernatant. repeat for a total of two washes.

Note: If the total volume of sample plus reagent exceeds 200 μl, then use a wash volume of at least the volume of sample plus reagent. A dry time is optional to ensure all traces of Ethanol are removed. For fragments 10 kb and larger, do not over dry the bead ring (bead ring appears cracked if over dried) as this will significantly decrease elution efficiency.

1. Remove the tube from the magnet plate, and then add proper amount of elution buffer/TE buffer/ddH2O to the tube and pipette mix 10 times. Incubate for at least 2 minutes at room temperature.
2. Place the tube onto a magnetic stand for 2 minutes to separate beads from the solution.
3. Transfer the eluate to a new 1.5 ml tube.

**ASDPS method**

1. Shake the BeaverBeadsTM Mag COOH-300 bottle to resuspend any magnetic particles that may have settled. Leave at room temperature for 30 min.
2. The magnetic beads were washed twice with distilled water, eg: add 100ul resuspended beads in the tube, place the tube on an appropriate magnetic stand to separate the beads from the supernatant, repeat for a total of two washes.
3. Add 550 μl buffer B (beads: buffer =1:5.5) in the tube and mix well (550ul beads-buffer).

| buffer B | Stock | 12.375mL |
| --- | --- | --- |
| PEG 8000 |  | 3.6g |
| NaCl | 5M | 7.2mL |
| MgCl2 | 2M | 293.4ul |
| ddH2O |  | volume to 12.375mL |

1. This step binds DNA fragments to the magnetic beads. Mix reagent and sample thoroughly by pipette mixing 10 times. Let the mixed samples incubate for 10 minutes at room temperature for maximum recovery. Eg: 60ul beads-buffers are added into 100ul DNA samples tubes, mix well and sit at 25 °C for 10 mins.
2. Place the tube on an appropriate magnetic stand to separate the beads from the supernatant. If necessary, quickly spin the sample to collect the liquid from the sides of the tube or plate wells before placing on the magnetic stand.
3. Add 200 μl of 80% freshly prepared ethanol to the tube while in the magnetic stand. Incubate at room temperature for 30 seconds, and then carefully remove and discard the supernatant. repeat for a total of two washes.

Note: If the total volume of sample plus reagent exceeds 200 μl, then use a wash volume of at least the volume of sample plus reagent. A dry time is optional to ensure all traces of Ethanol are removed. For fragments 10 kb and larger, do not over dry the bead ring (bead ring appears cracked if over dried) as this will significantly decrease elution efficiency.

1. Remove the tube from the magnet plate, and then add proper amount of elution buffer/TE buffer/ddH2O to the tube and pipette mix 10 times. Incubate for at least 2 minutes at room temperature.
2. Place the tube onto a magnetic stand for 2 minutes to separate beads from the solution.
3. Transfer the eluate to a new 1.5 ml tube.
