# Supplementary material for: An SPRI beads-based DNA purification strategy for flexibility and cost-effectiveness
Source: BMC Genomics. 2023 Mar 16;24:125. doi: 10.1186/s12864-023-09211-w (PMC10022144; doi:10.1186/s12864-023-09211-w)
Supplement: Supplementary file 3 — Additional file 3: Supplementary file 2. The original DNA Electrophoretic in the paper. [file 12864_2023_9211_MOESM3_ESM.docx]

**Additional file**

The original, uncropped DNA Electrophoretic in the main paper as follows:

**Fig.S2**


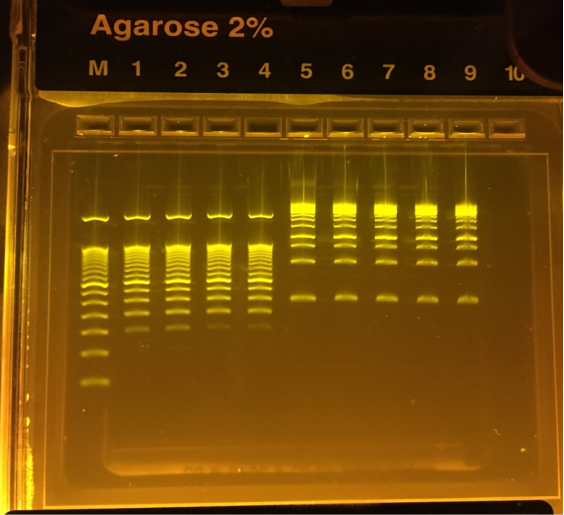
**A**

B


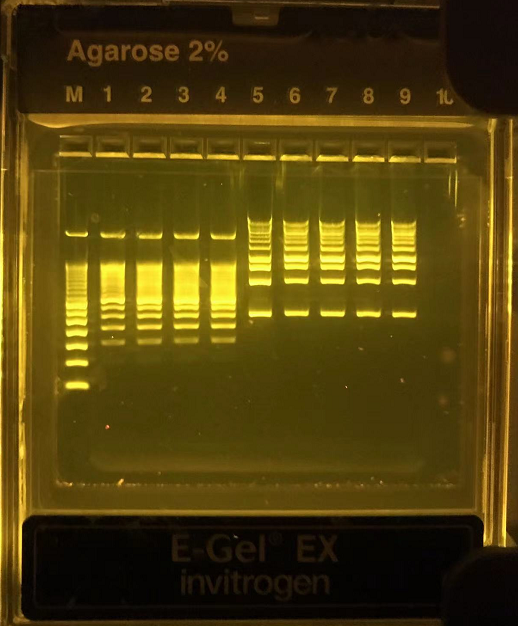


**Fig.S2** **A** The original, uncropped DNA Electrophoretic in the main paper Fig.2b;

**B** Repeated experiment, the original, uncropped DNA Electrophoretic.

Note: DNA electrophoresis was performed using E-gel (precast agarose gels), and pictures were taken using a cell phone, the same as below.

**Fig.S3**

**A**


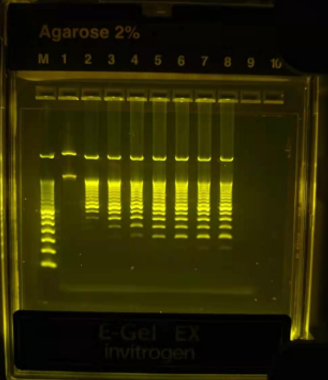


**
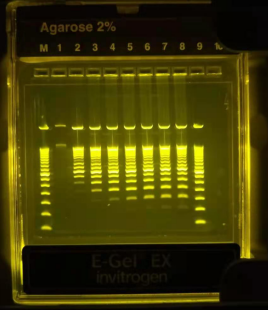
B**

**Fig.S3 A** The original DNA Electrophoretic in the main paper Fig.2d; B Repeated experiment, the original DNA Electrophoretic.

**Fig.S4**

**A**


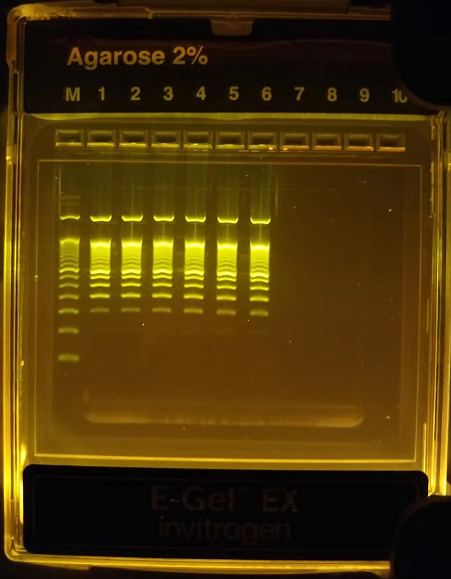


**B**


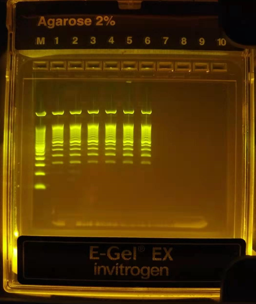


**Fig.S4 A** The original DNA Electrophoretic in the main paper Fig.3a; **B** Repeated experiment, the original DNA Electrophoretic.

**Fig.S5**

**A**


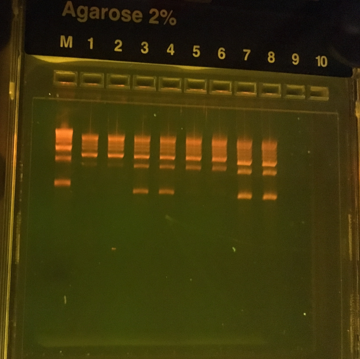


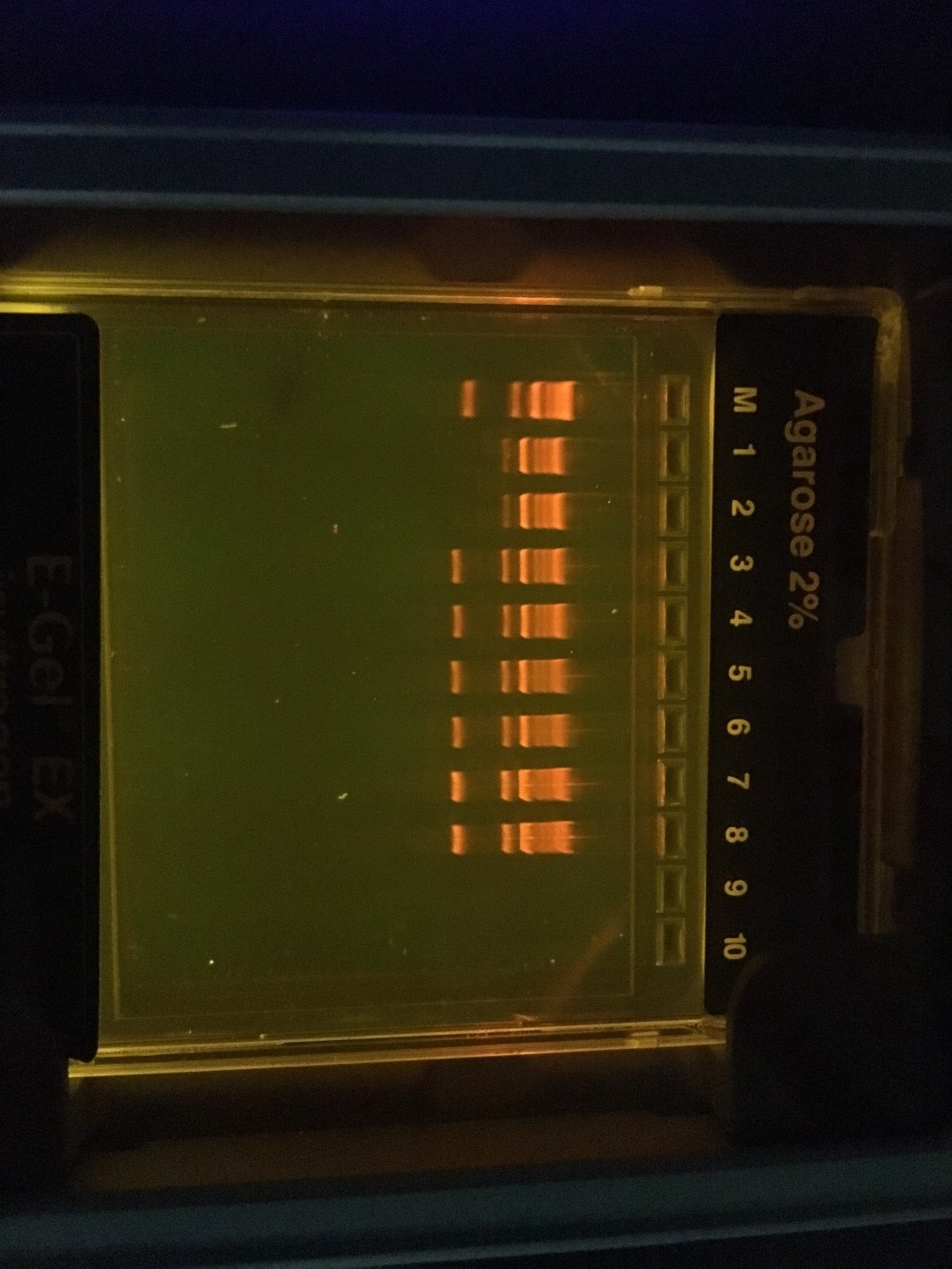


**B**

**C**


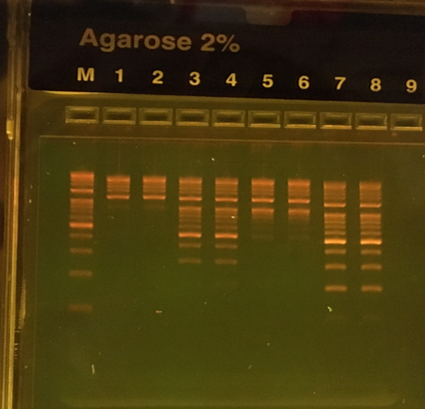


**Fig.S5 A** The original DNA Electrophoretic in the main paper Fig. S1B; **B** The original DNA Electrophoretic in the main paper Figure S1C; **C** The original DNA Electrophoretic in the main paper Figure S1D.
